# Foreign Correspondence

**Published:** 1838-02-28

**Authors:** 


### FOREIGN CORRESPONDENCE.

Paris, 25th Dec. 1837.

* * * On Wednesday, the 29th November, whilst going around the wards, a woman presented herself to M. Cazeau, at the Clinique of the Faculty. She was about four feet in height, aged twenty five, and so much deformed that it was determined instantly to make an examination. She was then in labour, and with her first child. M. Cazeau conducted her into a private apartment, where three or four of us only were admitted. We all examined her thoroughly, and came at once to the conclusion that it was impossible for the child to be delivered in the natural way. As had been surmised, the deformity involved the pelvis. The antero-posterior diameter did not exceed two inches. The child was alive, for I heard, (as all who were present did) the beating of the heart most distinctly.

A consultation having been called, some proposed the division of the symphisis pubis, and others the Cæsarean operation. The last was determined on, but, instead of its being undertaken immediately, they postponed it until the next morning at half past eight.

At the appointed hour the woman was brought into the amphitheatre. She was so much changed, and had sunk so low during the night, (for she had been thirty-six hours in labour,) that, had it not been for her deformity, I should not have recognized her. There were many surgeons and students present, and the operation was performed by M. Moreau, assisted by M. M. Merrel, Cazeau, and Dupol. The room was so much crowded that I could not get near enough to see very well, but the operation was described to me and seems to have been well enough performed. The child was either dead when removed, or died a minute or two after, almost necessarily from the length of time the mother was in labour. On the night following the day on which the operation was performed, (the night of the 30th,) *the mother also died.*

Far be it from me to censure the conduct of men who hold so high a rank in the profession, and although the result might not have been more favourable, still I cannot help thinking that the chances of recovery would have been much greater had the operation been performed earlier instead of waiting until a healthy woman, with apparently a good constitution, was reduced to so low a state as almost to preclude the possibility of its being attended with success.

Dr. Mott of New York, is still here, and has a good practice. * * * I have seen Dr. Warren, of Boston, also, who attends the hospitals as regularly as any of us. He leaves in a day or two for Italy. * * * *

